# Characterization of the Behavioral and Molecular Effects of Acute Exposure to the Fourth-Generation Synthetic Cannabinoid, 5F-EDMB-PICA, in Male Mice of Different Age Groups

**DOI:** 10.3390/ijms262110424

**Published:** 2025-10-27

**Authors:** Kaixi Li, Peng Xu, Yiming Wang, Xuesong Shi, Yuanyuan Chen, Simeng Zhang, Jingzhi Ran, Yanling Qiao, Yawen Xu, Yuan Pang, Bin Di

**Affiliations:** 1 School of Pharmacy, China Pharmaceutical University, Nanjing 210009, China; 2 Office of China National Narcotics Control Commission, China Pharmaceutical University Joint Laboratory on Key Technologies of Narcotics Control, Beijing 100193, China; dongwufang123@163.com; 3 Key Laboratory of Drug Monitoring and Control, Drug Intelligence and Forensic Center, Ministry of Public Security, Beijing 100193, China; 4 Biomanufacturing Center, Department of Mechanical Engineering, Tsinghua University, Beijing 100084, China

**Keywords:** adolescent, fourth-generation synthetic cannabinoids, memory, transcriptomics

## Abstract

Adolescents and young adults are using synthetic cannabinoids at increasing rates, and the use of synthetic cannabinoids (SCs) carries significant medical and psychiatric risks. Although studies have been conducted to preliminarily explore the pharmacological effects of the fourth-generation synthetic cannabinoid 5F-EDMB-PICA (ethyl 2-(1-(5-fluoropentyl)-1 H-indole-3-carboxamido)-3,3-dimethylbutyrate), there is still a lack of research addressing its deleterious effects in mice of different age groups. We investigated the effects of 5F-EDMB-PICA on multiple aspects of emotional functioning, locomotor performance, and cognitive functioning in adolescent and young adult mice by determining the affinity of 5F-EDMB-PICA for cannabinoid receptors in conjunction with behavioral experiments and transcriptomic analyses. Acute 5F-EDMB-PICA administration disrupted anxiety regulation, motor control, and spatial memory in mice of different age groups; accompanying hippocampal transcriptomic screens further pinpointed candidate genes that may mediate these deficits. This study establishes the molecular network of synthetic cannabinoid neurological harms, provides key gene expression profiles for subsequent in-depth analysis of the harm mechanisms, and also provides more data support for future control of cannabinoids.

## 1. Introduction

As described in the World Drug Report 2024, the limited data currently available suggest that synthetic cannabinoid receptor agonists (‘synthetic cannabinoids’) have become the most commonly used new psychoactive substances. And their recreational use has become a growing public health problem, with adolescents [1] and military personnel [2] being the most common users in Western societies [3]. Unlike Δ9-THC’s partial activation, synthetic cannabinoids behave as high-efficacy full agonists at both CB1 and CB2 sites, eliciting a toxicity profile that surpasses that of plant-derived cannabinoids [4]. As a case in point, acute toxicity caused by synthetic cannabinoids (SCBs) has been associated with an array of negative health impacts. These include rapid heart rate (tachycardia), increased blood pressure, disruptions in visual and auditory perception, constricted pupils (miosis), feelings of restlessness, anxious moods, seizure activity (convulsive episodes), difficulty breathing (dyspnea), and gastrointestinal issues like nausea and vomiting (emesis) [3]. The impact of synthetic cannabinoids on the nervous system is profound and concerning. They can induce a range of adverse effects, such as confusion, agitation, irritability, paranoia, disorganization, and anxiety [5]. With the emergence of newer generations of synthetic cannabinoids, these negative effects have become more severe. There has been a notable increase in the incidence of panic attacks, convulsions, and other serious health complications [6,7]. In summary, the risks posed by synthetic cannabinoids are significant and multifaceted, representing a considerable threat to both physical and mental health.

**Figure 1:**
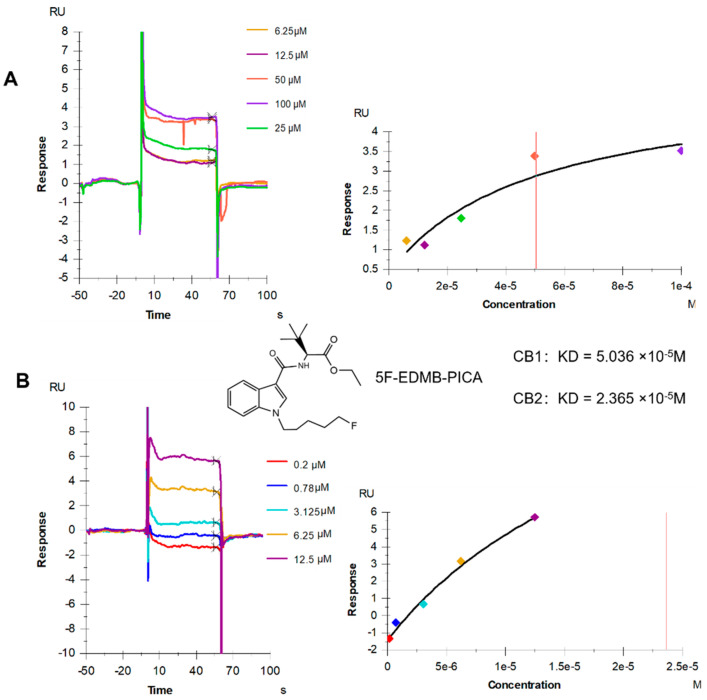
SPR measurements of 5F-EDMB-PICA binding to CB1 receptors (**A**) and CB2 receptors (**B**). In the binding and fitting plots, each color represents the corresponding sample concentration. The vertical red solid line in the fitted curve marks the KD value, which represents the concentration of the analyte when 50% of the binding sites are occupied.

Although illicit production of synthetic cannabinoids has declined since 2014 due to regulatory crackdowns and cost factors, the fourth-generation synthetic cannabinoid receptor agonists (SCRAs) from which they are derived, as a class of New Psychoactive Substances (NPS) that are the second-most-abused compounds with demonstrated potentiated pharmacological activity and potential toxicity, pose a serious challenge to public health due to their unknown mechanism of action and the fact that they are not yet under targeted control (only partially controlled through the World Health Organisation’s International Classification (ICC) in most countries. However, because of their unknown mechanism of action and the fact that they are not yet subject to targeted control in most countries (they are only partially controlled through the World Health Organisation’s (WHO) International Classification), they pose a serious challenge to public health. In September 2020, 5F-EDMB-PICA (ethyl 2-(1-(5-fluorophentyl)-1 H-indole-3-carboxamido)-3,3-dimethylbutyrate) (Figure 1) was analyzed for the first time by the Hungarian police.5F-EDMB-PICA mass spectrometry data were included in the Kelman Spectral Library in June 2020, and a seizure was made in 2021 by China of an herbal product containing 5F -EDMB-PICA [8]. It has been found to have a pronounced quadruple response characteristic of cannabinoid analogs and to produce significant psychoactive dependence at smaller doses than the first-generation synthetic cannabinoid JWH-018 [9]. In China, a whole class of synthetic cannabinoid substances has been listed since July 2021, although internationally, 5F-EDMB-PICA is temporarily not controlled under the United Nations Single Convention on Narcotic Drugs of 1961 and the Convention on Psychotropic Substances of 1971. Evidence for the compound’s additional hazards remains fragmentary, leaving a critical data gap that warrants further investigation.

As previously noted, the highest rates of SC use are observed among adolescents and young adults [1], which is especially alarming. Although there have been studies that have initially explored the pharmacological effects of the synthetic cannabinoid 5F-EDMB-PICA, there is still a lack of studies specific to its harmful effects in mice of different age groups. In this study, we revealed the effects of 5F-EDMB-PICA on anxiety, motor inhibition and impaired spatial memory in mice of different age groups by determining its affinity for cannabinoid receptors and combining it with behavioral experiments and hippocampal transcriptomic analyses, as well as providing a gene mapping resource, which provides basic data for the subsequent study of the mechanism of harm and also provides a theoretical basis for subsequent studies on the control of synthetic cannabinoid substances.

## 2. Results

### 2.1. Results of SPR

Fitting was conducted using the Affinity model in the Biacore T200 results analysis software (version: 3.2, serial number: BIA-0045, this software is licensed to: cytiva, Marlborough, MA, USA), with the obtained SPR signal curves shown in Figure 1. The affinity values were presented in Figure 1. The results showed that 5F-EDMB-PICA could interact with both CB1 (Figure 1A) and CB2 (Figure 1B) receptors, with 5F-EDMB-PICA having a higher affinity for CB2 (KD = 2.365 × 10^−5^ M) than CB1 (KD = 5.036 × 10^−5^ M).

### 2.2. Effects of 5F-EDMB-PICA on the Regulation of Locomotor Activity and the Induction of Anxiety-like Behaviors

**Figure 2:**
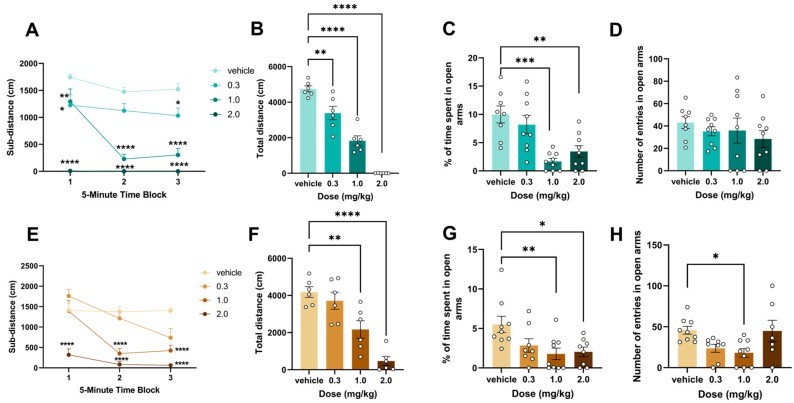
Effect of 5F-EDMB-PICA exposure during adolescence versus adulthood on locomotor activity and anxiety-like behaviors in mice. Panels (**A**–**D**) illustrate the impact of 5F-EDMB-PICA administration at doses of 0.3, 1, and 2 mg/kg on adolescent mice. Corresponding data for adult mice are presented in Panels (**E**–**H**). Each experimental group consisted of 6 –9 mice. All data are expressed as the mean ± standard error of the mean (SEM). Data significance is indicated as follows: * *p* < 0.05, ** *p* < 0.01, *** *p* < 0.001, **** *p* < 0.0001 (comparisons between the 5F-EDMB-PICA-treated groups and the vehicle control group). Data analyses were performed using two-way analysis of variance (ANOVA) with repeated measures for data in Panels (**A**,**E**), and one-way ANOVA for data in Panels (**B**–**D**,**F**–**H**).

Following the completion of preliminary affinity testing, we commenced behavioral testing, initially investigating spontaneous activity and anxiety-like behavior. According to the results of previous experiments [9], the ED_50_ value of the open-field test in ICR mice was 1.38 mg/kg after intraperitoneal injection of 5F-EDMB-PICA for 30 min, based on which the experimental doses were selected, two hours after i.p. administration of 5F-EDMB-PICA (0.3, 1 and 2 mg/kg), each mouse was sequentially placed in an open-field chamber and then on an elevated plus-maze for assessment of spontaneous activity and anxiety-like behavior in order to explore the locomotor activity and anxiety in mice of different age groups. The results showed that at 2 h after the injection of 0.3 mg/kg, the difference between the sub-distance and total distance of 5 min block in adolescent mice was statistically significant (F (3,20) = 66.87, *p* < 0.0001) (Figure 2A,B), and the difference between the distance of exercise in adult mice and that of the vehicle group was statistically significant at 2 h after the injection of 1.0 mg/kg (F (3,20) = 19.79, *p* < 0.0001) (Figure 2E,F). In the elevated plus maze, 1.0 mg/kg reduced open-arm dwell time relative to vehicle in both adolescent and adults (F (3,31) = 9.937, *p* < 0.0001)., (F (3,31) = 0.5942, *p* = 0.6235) (Figure 2C,G), and the adult mice entering the open arm was also significantly lower (F (3,29) = 4.144, *p* = 0.0146) (Figure 2H), which was not observed in adolescent mice (Figure 2D).

### 2.3. Behavioral Assessment Study Investigating the Effects of 5F-EDMB-PICA on Memory in Mice of Different Age Groups

**Figure 3:**
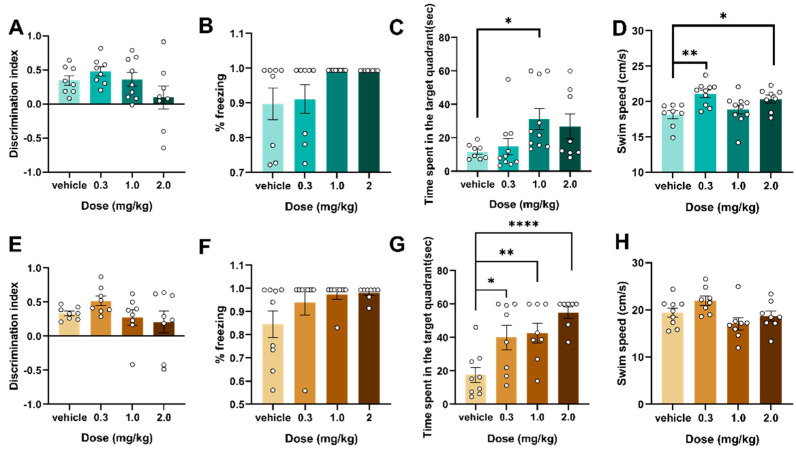
Effects of 5F-EDMB-PICA (administered at doses of 0.3, 1, and 2 mg/kg) on recognition memory (assessed via discrimination index), fear memory (measured as %freezing), spatial memory (evaluated by time spent in the target quadrant), and swim speed were examined in adolescent mice (see Figures (**A**–**D**)) and adult mice (see Figures (**E**–**H**)). Each experimental group consisted of 8–10 mice. All data are expressed as the mean ± standard error of the mean (SEM). Data significance is indicated as follows: * *p* < 0.05, ** *p* < 0.01, **** *p* < 0.0001 (comparisons between the 5F-EDMB-PICA-treated groups and the vehicle control group). Data analyses were performed using one-way ANOVA for data.

Following an initial exploration of behavioral models, we commenced research into memory across various levels. Two hours post-injection, spatial and recognition memory were compared across age groups using novel-object recognition, contextual fear conditioning and the Morris water maze; outcomes are displayed in Figure 3. In the new object recognition and fear memory extraction experiments, there were no significant changes in mice of different age groups (Figure 3A,B,E,F), and spatial navigation memory was significantly impaired in adolescent mice at a dose of 1 mg/kg (F (3,32) = 2.849, *p* < 0.05) (Figure 3C); meanwhile, swimming speed was significantly increased at 0.3 and 2.0 mg/kg (F (3,32) = 5.346, *p* < 0.05) (Figure 3D). In adult mice, there was a statistically significant difference at 0.3 mg/kg compared with the vehicle group (F (3,28) = 7.967, *p* = 0.05) (Figure 3G), with no significant change in swimming speed (Figure 3H). Also, the experimental results demonstrated that 5F-EDMB-PICA affected spatial memory navigation ability independent of locomotor ability (Figure 3D,H).

### 2.4. Global Hippocampal Transcriptional Response to 5F-EDMB-PICA

**Figure 4:**
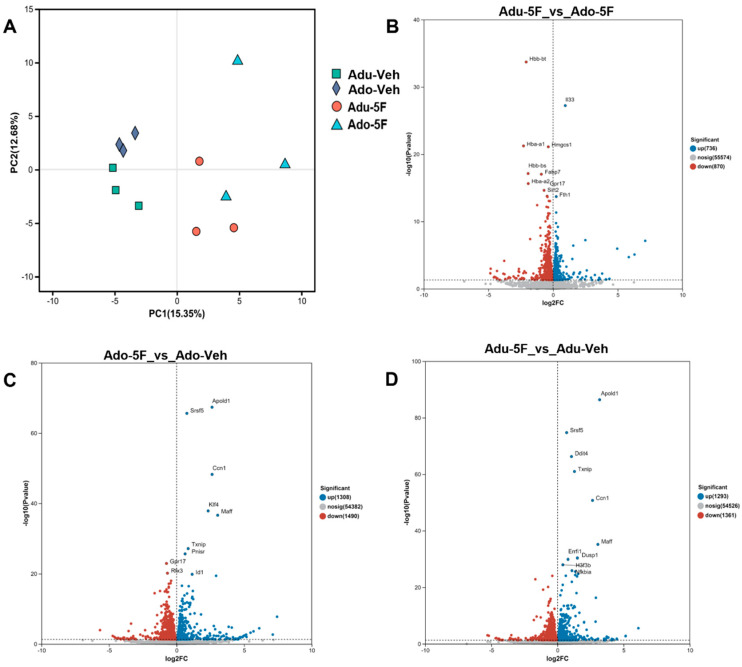
Overall impacts of 5F-EDMB-PICA exposure during adolescence and adulthood. (**A**) PCA of hippocampal RNA-seq profiles after vehicle or 5F-EDMB-PICA (2 mg kg^−1^, i.p.) in mice of different age groups. Volcano plots displaying differentially expressed genes (with *p* < 0.05) are shown for (**B**) comparisons between mice of different age groups at 2 h after 5F-EDMB-PICA exposure (2 mg/kg, intraperitoneal injection), as well as for (**C**,**D**) comparisons between 5F-EDMB-PICA-treated and vehicle control groups in adolescent and adult mice, respectively. Data analyses were conducted as detailed in Section 4.9.

Upon completion of the behavioral studies, the findings suggested that 5F-EDMB-PICA may exert a certain degree of influence on the hippocampus, the primary memory-forming structure. Based on this, we commenced an investigation into the underlying mechanisms. To map transcriptional consequences of 5F-EDMB-PICA across ontogeny, adolescent (Ado-5F) and adult (Adu-5F) mice received a single injection of the compound or vehicle (Ado-Veh, Adu-Veh); hippocampi were collected 2 h later for bulk RNA-seq. Principal-component analysis showed pronounced shifts in hippocampal gene-expression signatures relative to respective controls (Figure 4A). When comparing 5F-EDMB-PICA-exposed adolescent mice to their adult counterparts, 1606 genes exhibited differential expression, with 736 upregulated and 870 downregulated (Figure 4B). Relative to vehicle controls, adolescent mice treated with 5F-EDMB-PICA showed 2798 differentially expressed genes, consisting of 1308 upregulated and 1490 downregulated transcripts (Figure 4C). A comparable effect was observed in adult mice: 5F-EDMB-PICA administration led to alterations in 2654 genes compared to vehicle controls, with 1293 upregulated and 1361 downregulated (Figure 4D).

### 2.5. Functional Enrichment of Hippocampal Genes Disrupted by 5F-EDMB-PICA

**Figure 5:**
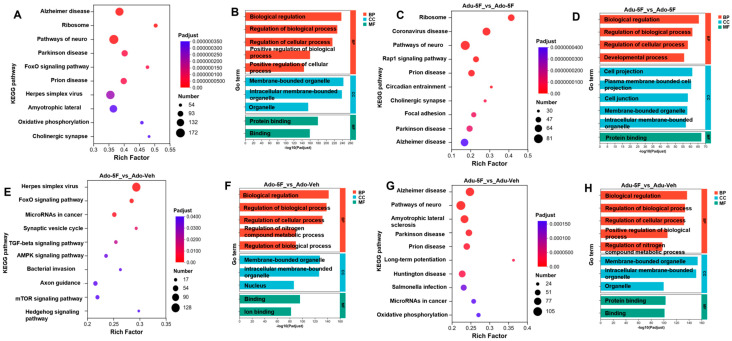
KEGG and GO Enrichment Analyses on the Age-Specific Effects of 5F-EDMB-PICA in Mice. Functional enrichment analyses were performed on all differentially expressed genes (DEGs). These DEGs were obtained from RNA sequencing (RNA-seq) data of hippocampal tissues—collected from mice of varying age groups that had been treated with either 5F-EDMB-PICA (at a dose of 2 mg/kg via intraperitoneal injection) or a vehicle control. Relevant results from these analyses are presented in Figure (**A**,**B**). Figure (**C**,**D**) display the KEGG pathways or GO terms (with adjusted *p*-value [Padj] < 0.05) associated with differentially expressed genes in mice of different age groups, respectively, at 2 h after 5F-EDMB-PICA administration (2 mg/kg, intraperitoneal injection). Corresponding KEGG pathways or GO terms (Padj < 0.05) for the vehicle control groups in adolescent and adult mice are presented in Panels (**E**–**H**). Data analyses were performed as outlined in Section 4.9. (Abbreviation notes for the figure: Pathways of neuro…: Pathways of neurodegeneration-multiple diseases; Herpes simplex virus…: Herpes simplex virus 1 infection; Amyotrophic lateral …: Amyotrophic lateral sclerosis; Bacterial invasion …: Bacterial invasion of epithelial cells).

For the identified specific genes, we further conducted functional enrichment analysis of hippocampal genes disrupted by 5F-EDMB-PICA. Transcriptomic analysis uncovered notable disruptions in biological pathways, as validated through Kyoto Encyclopedia of Genes and Genomes (KEGG) pathway analysis and Gene Ontology (GO) enrichment analysis. After applying the Benjamini–Hochberg correction to adjust *p*-values, a threshold of <0.05 was used to determine statistical significance. Through this analysis, 10 KEGG pathways and 10 GO terms showing significant enrichment were detected. These GO terms, in particular, spanned the three core ontological classifications—biological process (BP), molecular function (MF), and cellular component (CC)—as depicted in Figure 5.

Our analytical process commenced with a thorough, transcriptome-wide evaluation of all collected samples. When examining the pathways with the strongest enrichment signals (as shown in Figure 5A), three key pathways stood out: “Alzheimer’s disease” (with an adjusted *p*-value [Padj] of 3.63 × 10^−9^), “Ribosome” (Padj = 3.78 × 10^−9^), and “Pathways of neurodegeneration-multiple diseases” (Padj = 4.64 × 10^−9^). These findings further confirmed the preserved nature of learning and memory functions in the hippocampal region, as well as the indispensable functional role of these cognitive processes.

Additionally, genes transcribed across all samples exhibited robust enrichment in specific GO terms: within the BP category, this included “Biological regulation” (GO: 0065007, Padj = 2.069 × 10^−238^) and “Regulation of biological process” (GO: 0050789, Padj = 2.474 × 10^−228^); in the CC category, enrichment was observed for “Membrane-bounded organelle” (GO: 0043227, Padj = 1.775 × 10^−243^); and within the MF category, significant enrichment was detected for “Protein Binding” (GO: 0005515, Padj = 2.878 × 10^−180^) (Figure 5B). Next, we analyzed the distinct functional alterations in two experimental groups—adolescent mice (designated as Ado-5F) and adult mice (designated as Adu-5F)—following their exposure to 5F-EDMB-PICA. Our findings revealed that the Ribosome pathway was significantly enriched in the adolescent mouse group, with an adjusted *p*-value (Padj) of 1.03 × 10^−24^ (see Figure 5C). Further analysis of the hippocampal tissue from Ado-5F mice showed that, among the top-ranked GO terms for biological process (BP), the primary term focused on biological regulation (GO:0065007, Padj = 2.36 × 10^−66^); for molecular function (MF), the enrichment was dominated by genes related to protein binding (GO:0005515, Padj = 5.51 × 10^−68^); and for cellular component (CC), the enriched term corresponded to cell-projection components (GO:0042995, Padj = 5.17 × 10^−62^). These results are presented in Figure 5D. Compared with the vehicle group (Ado-Veh), we identified some interesting signaling pathways such as herpes simplex virus 1 infection (Padj 6.78 × 10^−18^) in the Ado-4F mouse gene (Figure 5E). Meanwhile, in GO enrichment analysis, biological regulation (GO: 0065007, Padj 1.19 × 10^−132^) was significantly enriched in BP, membrane-bounded organelle (GO: 0043227, Padj 1.46 × 10^−128^) in CC and binding (GO: 0005488, Padj 6.14 × 10^−97^) in MF (Figure 5F).

We similarly investigated the signaling pathways associated with KEGG and GO enrichment in adult mice (Adu-5F) compared to the vehicle group (Adu-veh). Results of the KEGG signaling pathway in adult mice exposed to 5F-EDMB-PICA showed significant enrichment mainly in “Alzheimer’s disease” (Padj 1.07 × 10^−8^) and “Pathways of neurodegeneration-multiple diseases” (Padj 2.21 × 10^−7^) (Figure 5G). Accompanied in the GO enrichment analysis, the biological regulation (GO: 0065007, Padj 1.18 × 10^−138^) was significantly enriched in BP, membrane-bounded organelle (GO: 0043227, Padj 2.22 × 10^−154^) in CC and binding (GO: 0005488, Padj 6.45 × 10^−102^) in MF (Figure 5H)

### 2.6. 5F-EDMB-PICA-Evoked Hippocampal Gene-Expression Changes

**Figure 6:**
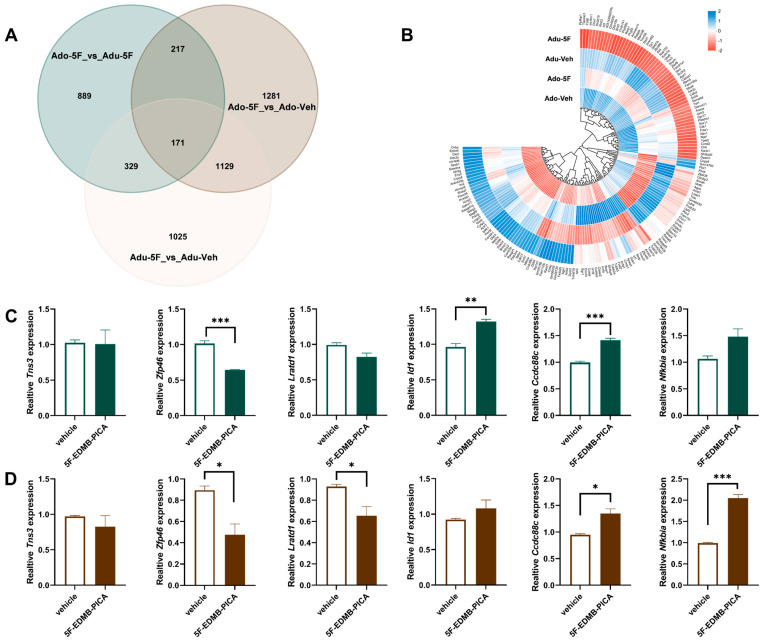
Gene Expression Variations in Adolescent versus Adult Mice Exposed to 5F-EDMB-PICA. (**A**) Venn diagram was employed to analyze the gene set distributions across distinct experimental groups. (**B**) Heatmap illustration of transcriptional changes in 171 overlapping genes, comparing adolescent and adult mice treated with 5F-EDMB-PICA or the corresponding vehicle. In the adolescent (**C**) and adult (**D**) mice groups, 5F-EDMB-PICA (2 mg/kg, i.p.) and vehicle control were administered to distinct subgroups, respectively. A two-hour interval following treatment elapsed before hippocampal tissues were harvested; subsequently, the expression of specific target genes in these tissues was measured via qRT-PCR. Data assessments were conducted in accordance with the protocol detailed in Section 4.9. All data are presented as mean ± SEM. * *p* < 0.05, ** *p* < 0.01, *** *p* < 0.001 (n = 3 per group).

Finally, we investigated the changes in hippocampal-specific gene expression induced by 5F-EDMB-PICA. Figure 6A presents a Venn diagram utilized for analyzing the gene counts within each target gene set, as well as the overlapping patterns among these sets. Through systematic identification of overlapping genes across groups, we identified a total of 171 genes that were commonly present in mice of different age groups.

We next clustered the 171-member gene set (Figure 6B) to visualize their expression signatures. Guided by the initial KEGG and GO enrichment profiles, we shortlisted candidates with the largest expression shifts after 5F-EDMB-PICA in both age cohorts and validated them by qPCR. The verified genes included *Tns3* (tensin 3), *Zfp46* (zinc finger protein 46), *Lratd1* (LRAT domain containing 1), *Id1* (Inhibitor of DNA Binding 1), *Ccdc88c* (coiled-coil domain containing 88C), and *Nfkbia* (NFKB Inhibitor Alpha), with detailed results shown in Figure 6C,D.

Specifically, in adolescent mice exposed to 5F-EDMB-PICA, significant alterations were detected in the expression of the *Zfp46*, *Id1*, and *Ccdc88c* genes (Figure 5C). In contrast, adult mice exposed to the same substance displayed statistically significant differences in the *Zfp46*, *Lratd1*, *Ccdc88c*, and *Nfkbia* genes (Figure 5D). Based on these observations, we proposed that the *Zfp46* and *Ccdc88c* genes may act as key target genes contributing to tissue injury in mice of different age groups.

Collectively, these results demonstrate that a single exposure to the synthetic cannabinoid 5F-EDMB-PICA induces changes in motor function, anxiety-related behaviors, and spatial navigation capabilities. Additionally, this exposure exerts extensive impacts on the expression of key genes within the hippocampal tissue of the brain.

## 3. Discussion

In this study, we systematically analyzed the effects of 5F-EDMB-PICA on anxiety in mice of different age groups using a multivariate approach of cannabinoid receptor affinity assays, behavioral phenotyping, and hippocampal transcriptome analysis. The effects of 5F-EDMB-PICA on anxiety-like behavior, inhibition of motor function, and anxiety in adolescent and young adult mice were systematically analyzed. Our findings demonstrate that 5F-EDMB-PICA binds to both CB1 and CB2 receptors with measurable affinity. Following administration, this compound induced notable inhibitory effects on behavior and motor function, while also impacting learning and memory processes, in the hippocampal tissue of adolescent mice and young adult mice. To further explore these effects, we performed RNA sequencing on hippocampal samples, followed by quantitative polymerase chain reaction (qPCR) validation and bioinformatics analysis. These experiments led to the identification of an age-specific gene expression profile linked to the pharmacological actions of the receptors. Importantly, this profile revealed that 5F-EDMB-PICA substantially altered the specific expression patterns of the *Zfp46* and *Ccdc88c* genes. 5F-EDMB-PICA acts as a fourth-generation synthetic cannabinoid, an indole-based cannabinoid receptor agonist containing an ethyl dimethyl butyrate (EDMB) linking group, a carboxamide linker, and a 5-fluoropentyl chain. It is structurally related to 5F-MDMB-PICA (listed in Schedule II of the Convention on Psychotropic Substances of 1971 in 2020) [10]. Based on this, we first determined the affinity of 5F-EDMB-PICA for cannabinoid receptors (both CB1 and CB2 receptors) using the SPR technique, and our results showed that it has a strong affinity for both CB1 and CB2 receptors, and its affinity for CB2 is stronger than that for CB1 receptors. Interestingly, similar results were also found in the receptor affinity test of 5F-MDMB-PICA: the EC_50_ of CB1/CB2 was 3.26 nM/0.87 nM, respectively, with a selectivity ratio of about 3.75-fold [11], which we speculate may be achieved through the formation of hydrogen bonding of the indazole-3-carboxamide backbone with key amino acids in the transmembrane region of the CB2 receptor and the use of the 5-fluorine substituents for enhanced hydrophobic interactions with the 5-fluorine substituent to achieve preferential activation of CB2 [12]. Despite being more selective for CB2, its equally activated CB1 receptor was able to centrally mediate psychoactive side effects, which was further confirmed by the results of subsequent behavioral experiments in our study.

As far as we are aware, this is the first head-to-head evaluation of the acute impact of the synthetic cannabinoid 5F-EDMB-PICA in mice of different age groups. Previous studies in ICR mice found an LD_50_ of 1.38 mg/kg for locomotor depression [9], and our experiments built on this to determine the experimental dose, with the maximum dose chosen to be 2 mg/kg, with larger doses resulting in significant locomotor depression affecting subsequent cognitive-behavioral measurements and resulting in false positives. The results showed that adolescent mice induced significant motor inhibition at a much smaller dose of 0.3 mg/kg compared to adult C57BL/6 mice, again demonstrating that human adolescence is characterized by heavy use of recreational drugs, including at the experimental level [13]. Meanwhile, we examined anxiety-like behaviors by means of an elevated cross maze, where initial exposure of both adolescent and adult mice resulted in significant differences at a dose of 1.0 mg/kg, and our results are supported by the results of behavioral experiments with the analog 5F-MDMB-PICA, according to findings from Aurora Musa et al., adult mice that received repeated treatment with 5F-MDMB-PICA during adolescence displayed increased burying activity in the marble burying test. This specific behavioral phenotype is widely considered a marker that reflects heightened anxiety in experimental subjects [14].

In the next behavioral experiments on the effects of cognitive functioning, we found no significant changes in memory for recognition of new objects as well as fear memory extraction, and only spatial navigational memory showed impairments in experiments with the water maze, a result that deserves to be discussed in more depth. It has been reported that the administration of synthetic cannabinoids resulted in a significant decrease in the proportion of time that animals spent exploring new objects, suggesting diminished situational memory recognition. This effect may be related to the blockage of postsynaptic signaling due to CB1 receptor activation in the hippocampus [15]. As well as in a conditioned fear memory task, the synthetic cannabinoid HU-210 reduces the animals’ stiffness reaction time to the conditioned stimulus, suggesting impaired acquisition of fear memory. This effect is associated with inhibition of glutamatergic transmission by amygdala CB1 receptor activation [16]. In Morris-water-maze tests, a single intravenous shot of Δ9-THC (8 mg/kg) before training selectively blocked spatial acquisition without changing swim speed [17], whereas the synthetic agonist WIN55212-2 (1–3 mg/kg, systemic) produced a comparable learning deficit [18]. Our data with 5F-EDMB-PICA mirror these findings—impaired navigation in both adolescent and adult mice—while locomotor activity remained intact, indicating a memory-specific effect rather than a motor confound. We hypothesized that the differences in cognitive deficits were related to the affinity of CB1 and CB2 receptors, as CB2 receptors are less centrally expressed and a single administration of CB2 would have a weaker effect on cognitive function or even on cognitive deficits due to CB1 activation, whereas CB2R activation was reportedly ineffective for hippocampal-dependent spatial cognitive dysfunction in the Morris water maze test (*p* > 0.05) [19]. The behavioral findings align with the outcomes of affinity assays conducted in surface plasmon resonance (SPR) experiments: specifically, stronger affinity for the CB2 receptor was associated with reduced impairment of cognitive function. However, it is important to note that the tests employed in this study did not enable us to evaluate other aspects of the cognitive domain—such as impulsivity and attention—and this limitation cannot be overlooked. Given this constraint, coupled with the inconsistent results reported in previous studies investigating the impacts of synthetic cannabinoids (SCs) on cognitive functions, we cannot exclude the possibility that 5F-EDMB-PICA might induce adverse effects on cognitive processes.

Since CB1 [20] and CB2 receptors [21] have a high density of expression in the hippocampus, and the hippocampus serves as a major acting brain region, dominating learning and memory [22]. Two hours after administering a single intraperitoneal (i.p.) dose of 5F-EDMB-PICA (2 mg/kg) to mice grouped by age, hippocampal tissues were isolated from these age-stratified animals. Subsequently, bulk RNA sequencing (bulk RNA-seq) was used to characterize the age-specific transcriptional response profiles in the isolated hippocampus. Exposure to 5F-EDMB-PICA, a synthetic cannabinoid, triggers complex alterations in multiple signaling pathways. KEGG pathway analysis reveals a striking age-specific divergence in the biological responses of adolescent and adult mice to this compound. In adult mice, the significantly enriched pathways are predominantly associated with neurodegenerative disorders, including Alzheimer’s disease, Parkinson’s disease, and other neurological conditions. This indicates that 5F-EDMB-PICA may disrupt central nervous system homeostasis, potentially accelerating the progression of neurodegenerative processes. Conversely, adolescent mice exhibit notable activation of pathways related to ribosome function and viral infection responses, such as those associated with coronavirus disease and herpes simplex virus infection. We then re-validated in q-PCR the genes found from the 171 intersecting genes clustering analysis—with initial concordance of expression after drug administration—and found that the *Zfp46* and *CCdc88c* genes serve as important targets that can co-alter the expression of the molecules in the hippocampal tissues of both adolescence and adulthood. The *Zfp46* gene encodes a protein belonging to the zinc finger family of proteins predicted to have DNA-binding transcriptional repressor activity specific to RNA polymerase II and to be involved in the negative regulation of RNA polymerase II transcription. The *Ccdc88c* gene encodes the coiled-coil domain-containing protein 88C (*Ccdc88c*). This gene plays a critical role in mediating cell communication processes that occur during neural development. *NFkbia* encodes NF-κB inhibitor α, which suppresses activation of the NF-κB pathway. Both CB1 and CB2 receptors regulate NF-κB activity via G protein-coupled signaling; high-affinity agonists can upregulate *NFkbia* expression to enhance anti-inflammatory effects. This finding is corroborated by the high-affinity CB1 and CB2 interactions observed in SPR experiments within this study. Hippocampal CB1 receptors interact with TrkB (Tropomyosin Receptor Kinase 4), wherein CB1 high-affinity agonists enhance TrkB phosphorylation. This activates the downstream PI3K/Akt pathway, thereby regulating the expression of Tns3, a gene associated with synaptic remodeling [23]. Our transcriptomic findings further elucidate the causal relationship between high-affinity cannabinoid receptors and the transcriptome. We propose that these genes should be prioritized in subsequent studies to investigate the mechanisms underlying memory impairments in adolescence and adulthood following acute exposure to 5F-EDMB-PICA, necessitating more in-depth research.

Additionally, transcriptomic experiments indicated involvement of pathways associated with herpes simplex virus type 1 infection, amyotrophic lateral sclerosis, and bacterial invasion of epithelial cells within KEGG-enriched signaling pathways. These findings suggest another phenomenon warrants attention: Ferrarini EG et al. [24] reported that broad-spectrum cannabis oil ameliorates guanethidine-induced fibromyalgia in mice. García-Toscano L et al. [25] identified the neuroprotective role of cannabidiolic acid in TDP-43 transgenic mice (an ALS experimental model), while Cortes-Justo E [26] investigated how cannabidiol oil delays pancreatic dysfunction in Wistar rats fed a high-calorie diet. De Freitas A et al. [27] reported cannabinoids as potential modulators of the human parasite Trichomonas vaginalis and as cytotoxic agents. These studies corroborate our findings, suggesting that their potential as beneficial developmental agents may also warrant future consideration.

It is worth noting that our experiment has certain limitations, as the influence of gender factors was not addressed in this study, but should not be overlooked in future research. The existing literature reports that following repeated administration of the synthetic cannabinoid AKB48 in both male and female mice, distinct differences exist in the neuroplasticity of CB1 receptors within the cortex, as well as in the pharmacotoxicological effects and pharmacokinetics [28]. Cristina Izquierdo-Luengo et al. reported that adolescent exposure to the synthetic cannabinoid JWH-018 induces persistent neurobiological alterations associated with psychosis-like symptoms, exhibiting a gender-dependent pattern [29]. Furthermore, in mouse models, the synthetic cannabinoid MMB-Fubinaca elicited more pronounced behavioral and electrophysiological effects in male mice, with these effects manifesting more markedly in a CB1 receptor-dependent manner [30]. These findings suggest that synthetic cannabinoids elicit gender-specific responses, highlighting the need to include female subjects in future studies to address this research gap.

This study not only elucidates for the first time the age-related neurobehavioral deficits induced by 5F-EDMB-PICA during developmental stages, but also integrates receptor pharmacology, behavioral phenotyping, and transcriptomic data to construct a molecular network of cannabinoid-induced neurotoxicity. This provides a critical gene expression resource for further elucidating the damage mechanisms and establishes an experimental foundation for developing cannabinoid risk management strategies tailored to the physiological characteristics of different age groups in mice. Furthermore, it holds significant translational implications for human health protection, clinical risk early warning, and public health strategy formulation. Adolescents should be prioritized as the core population for cannabinoid abuse prevention and control. Clinicians encountering adolescents presenting with cognitive impairment or emotional disturbances should proactively inquire about synthetic cannabinoid exposure to prevent misdiagnosis of ‘occult neurotoxic injury’. Transcriptional abnormalities in hippocampal *Tns3* (regulating synaptic adhesion) and *Nfkbia* (inhibiting the NF-κB inflammatory pathway) may provide targets for developing ‘adolescent cannabinoid injury repair drugs.’ Current clinical diagnosis of synthetic cannabinoid poisoning relies primarily on history and symptoms (e.g., agitation, hallucinations, memory impairment), lacking objective biomarkers. The identified association between elevated transcription levels of *CCdc88c* and *Id1* with memory impairment may enable future rapid clinical diagnosis of synthetic cannabinoid neurotoxicity by detecting peripheral blood expression of these genes (or their encoded proteins). This approach is particularly suitable for patients unable to articulate clear medical histories, such as adolescents or those with impaired consciousness. From a clinical perspective, these findings not only provide experimental support for risk prevention and diagnostic biomarker development concerning synthetic cannabinoid abuse among adolescents but also offer a more comprehensive theoretical basis and practical intervention strategies for addressing synthetic cannabinoid-induced cognitive impairment.

## 4. Materials and Methods

### 4.1. Animals

Adolescent and young adult male C57BL/6 mice were used in this study, with the former aged 28–35 postnatal days (PND) and the latter 49–56 PND [31]. All animals were sourced from SPF Biotechnology Co., Ltd. (Beijing, China).

The mice were housed in a controlled rearing environment, where the temperature was maintained at 25 ± 2 °C and humidity at 50 ± 10%, accompanied by a 12 h dark-light cycle (with lights turned on at 8:00 a.m.). Food and water were supplied ad libitum by SPF Biotech Co., Ltd. (Beijing, China). Prior to housing, the mice were randomly allocated based on their body weight, with 5 mice per cage. Each cage had dimensions of 466 × 314 × 200 mm, and the bottom was lined with dust-free, non-toxic corn cob bedding. This bedding was replaced twice a week to ensure a dry and clean living environment for the mice.

Consistent with the recommendation by Sorge et al. [32], all mouse handling procedures—including weighing, surgical operations, behavioral tests, and sacrifice—were exclusively carried out by female experimenters.

### 4.2. Drugs

5F-EDMB-PICA (purity 99%) was obtained from China Pharmaceutical University. First, the substance was dissolved in ethanol; subsequently, it was diluted to the target volume using a mixture of 5% Tween 80 and physiological saline (0.9% sodium chloride). In addition, solvents devoid of components were prepared, comprising ethanol, Tween 80, and saline. Prior to experimentation, all drug solutions were freshly prepared and administered via intraperitoneal (i.p.) injection.

### 4.3. Surface Plasmon Resonance (SPR)

#### 4.3.1. Experimental Reagents and Instruments

Reagents and Instruments: CB1, CB2 protein (prepared by China Pharmaceutical University); dimethyl sulfoxide (DMSO, Sigma-Aldrich (Marlborough, MA, USA), analytical grade); CM5 sensor chips, PBS-P buffer, amine-coupling kit, sodium acetate solutions (all from Cytiva, Marlborough, MA, USA); Biacore T200 instrument (Cytiva, USA) [33].

#### 4.3.2. Experimental Procedure

The CM5 chip was activated with EDC-NHS and then coupled with CB1, CB2 protein (15 μg/mL, 30 μg/mL in 10 mM sodium acetate, pH 4.0). Remaining active sites were blocked with 1 M ethanolamine-HCl. Test compounds were dissolved in DMSO and diluted in PBS-P buffer (5% DMSO), with a maximum concentration of 100 μM, followed by serial twofold dilutions. The data were processed using Biacore T200 result analysis software (Biacore T200 Evaluation Software, V3.2) [34].

### 4.4. Locomotor Activity

To evaluate the influence of 5F-EDMB-PICA on mice’s locomotor activity, spontaneous movement of adolescent and adult mice was analyzed at 2 h post-administration, with each testing session lasting 15 min [35].

Data collection was carried out using an automated locomotor activity testing system, which was supplied by Beijing Zongshi DiChuang Science and Technology Development Co., Ltd., located in Beijing, China. This system comprises a core experimental chamber, a high-definition camera, a data-processing computer, and a trajectory tracking module, which together enable the recording of mice’s total distance of spontaneous locomotion.

Each individual mouse was tested in a dedicated opaque Plexiglas chamber (35 cm × 35 cm × 35 cm), and this testing chamber was further placed inside a larger ventilated collection box with dimensions of 55 cm × 45 cm × 55 cm to ensure a stable testing environment.

### 4.5. Elevated Plus Maze (EPM)

An adapted version of the elevated plus maze (EPM), originally described in reference [36], was employed to gauge anxiety-related behaviors in mice. The experimental device used in this study was produced by Beijing Zongshi DiChuang Science and Technology Development Co., Ltd. (Beijing, China). This apparatus was composed of a black cross-shaped maze, which was placed at a height of 30 cm above the floor of the laboratory. The maze structure included a 5 × 5 cm central platform, from which four arms (each measuring 16 × 5 cm) extended radially. Of these four arms, two were classified as “closed arms” due to the presence of vertical enclosing walls, while the remaining two were designated “open arms” with unobstructed, open edges.

Two hours prior to the EPM test, each mouse received an intraperitoneal injection of either 5F-EDMB-PICA (at doses of 0.3, 1, or 2 mg/kg) or a vehicle control solution. During the 5 min testing period, mouse movements within the maze were monitored in real time using a combined camera-computer system integrated with automatic trajectory-tracking software (also supplied by Beijing Zongshi DiChuang Science and Technology Development Co., Ltd.). The system’s method for collecting and recording behavioral metrics draws upon Li et al. [37].

### 4.6. Novel Object Recognition Test (NOR)

Mice were subjected to the object recognition test, which took place in a square arena with low illumination; the arena had specific dimensions of 40 × 40 × 30 cm. Prior to the start of the task, mice received an intraperitoneal injection of 5F-EDMB-PICA, with a 2 h interval between injection and the initiation of testing. Mouse training, testing methods and calculation basis for indicators Li et al. [37].

### 4.7. Contextual Fear Memory

Specifications for the conditioned fear apparatus are detailed in the report by Li et al. [37]. For the training phase, each mouse is first exposed to the chamber context for 5 min; subsequent to this context exposure, five pairings of a tone (30 s in duration, 5 kHz frequency, 85 dB intensity) and a foot shock (2 s in duration, 0.6 mA intensity) are administered. The testing phase takes place 24 h after the training phase: two hours prior to testing, each mouse receives an intraperitoneal injection of either 5F-EDMB-PICA or the vehicle control. During the 3 min test session, a 30 s tone is played, and the freezing behavior exhibited by the mice throughout this tone period is recorded as a metric to assess fear memory recall.

### 4.8. Morris Water Maze

The experimental apparatus, training methods and test content were based on the research by Li et al. [37]. Mice underwent four randomized training sessions daily, starting from different quadrants. In each trial, mice were given 120 s to find the concealed platform. If they failed to locate it independently, they were directed to the platform and kept there for 15 s. A full 24 h after the training session, the mice’s memory retention ability was evaluated. Mice received an intraperitoneal injection of 5F-EDMB-PICA or solvent two hours pre-test.

### 4.9. RNA Sequencing (RNA-Seq)

In a separate cohort of experimental mice, hippocampal tissues were harvested at 2 h post-injection of either 5F-EDMB-PICA or its corresponding vehicle. This experimental procedure was conducted with two rounds of replicate validation in the form of parallel groups, and each group was supplemented with 3 parallel experimental subgroups. Total RNA was isolated from tissue slices with MJZol reagent (Meiji Biotech, Shanghai, China) following a standard Trizol protocol, and the resulting samples were transferred to Majorbio (Shanghai, China) for downstream workflows.

Next, the quality of the RNA samples was evaluated using the 2100 Bioanalyser (Agilent Technologies, Santa Clara, CA, USA), while the ND-2000 Spectrophotometer (NanoDrop Technologies, Wilmington, DE, USA) was utilized to perform quantitative analysis of the RNA. Only high-purity RNA samples that met the quality standards were selected for the preparation of sequencing libraries.

The construction of the RNA-seq transcriptome library was initiated with 1 μg of total RNA as the starting material, and the process strictly followed the operating instructions provided in the TruSeq™ RNA Sample Preparation Kit (Illumina, San Diego, CA, USA). After the library was quantified using the TBS380, the constructed paired-end RNA-seq sequencing library was subjected to sequencing analysis on the Illumina HiSeq Xten/Nova Seq 6000 sequencing platform (San Diego, CA, USA), with a read length set to 2 × 150 bp.

### 4.10. Quantitative Real-Time Polymerase Chain Reaction (qPCR)

Hippocampal tissues from mice were used as the source for total RNA purification, a process carried out with the RNAfast200 extraction kit (manufactured by Fastagen, Shanghai, China). Following purification, the concentrations of the isolated total RNA were determined using a NanoDrop spectrophotometer (supplied by Thermo Fisher Scientific, Darmstadt, Germany). One microgram of RNA was converted to cDNA with AdvanceFast SuperMix (YEASEN, Shanghai, China), and transcript levels were measured on a CFX96 platform (Bio-Rad, Hercules, CA, USA) using SYBR GREEN I Master Mix (cat. 11184ES08, YEASEN). Glyceraldehyde-3-phosphate dehydrogenase (GAPDH) served as the internal reference gene for normalization (primer sequences provided in the Table 1). Each reaction was run in triplicate with a total volume of 25 μL, containing 2 μL of the synthesized cDNA.

### 4.11. Data Analyses

Data processing and data evaluations were performed using GraphPad Prism 9 software (GraphPad Software, Inc., La Jolla, CA, USA). Data were analyzed with Student’s t-test, one-way ANOVA plus Holm–Sídak post hoc comparisons, or two-way ANOVA followed by Sídak’s test, chosen according to the experimental structure. All results were expressed as mean ± standard error of the mean (SEM). Data significance was denoted as follows: * *p* < 0.05, ** *p* < 0.01, *** *p* < 0.001, and “n.s.” (representing “not significant”) for non-significant differences.

## 5. Conclusions

In this study, researchers comparatively analyzed the neurobehavioral deficits induced by 5F-EDMB-PICA (a fourth-generation synthetic cannabinoid) in male mice belonging to distinct age groups. Moreover, by combining three key types of data—receptor affinity results, multiple behavioral assays, and transcriptomic information—the study constructed a molecular network map that delineates how cannabinoids cause neurological harm. This work not only provides a critical gene expression resource for subsequent studies investigating the mechanisms of memory impairment but also, by considering the physiological characteristics specific to different age cohorts, establishes an experimental basis for developing risk management strategies related to synthetic cannabinoids.

## Figures and Tables

**Table 1:** Primers used for qCR.

Genes	Sequence
*Tns3* forward	5′-CCCAGAGTCTGTGGAGTGTG-3′;
*Tns3* reverse	5′-AGGAATACTTGCAGGCTCGG-3′
*Zfp46* forward	5′-ACAGACCAGGAGGAGACGAA-3′;
*Zfp46* reverse	5′-GAGTCCCAGACATGAGCAGG-3′;
*Lratd1* forward	5′-AAGGAGTTTGGGGTTTCGGG-3′;
*Lratd1* reverse	5′-CCAGCAACTGAGATCCGTGATT-3′;
*Id1* forward	5′-GAACCGCAAAGTGAGCAAGG-3′;
*Id1* reverse	5′-GGAACACATGCCGCCTCA-3′;
*Ccdc88c* forward	5′-GGCTCATATCCAGGAGTTGATCTT-3′;
*Ccdc88c* reverse	5′-CTCGGTGGCAGCTTGTTTC-3′;
*Nfkbia* forward	5′-GCCAGCTGACCCTGGAAAAT-3′;
*Nfkbia* reverse	5′-CATCATAGGGCAGCTCATCC-3′;

## Data Availability

Data are contained within the article. All original data produced in the course of this study is openly available to any interested researchers or users. Interested parties can obtain this dataset by accessing the NCBI database, where it has been deposited under the unique Accession number: PRJNA1278960.
